# Association between plasma polyunsaturated fatty acids and depressive among US adults

**DOI:** 10.3389/fnut.2024.1342304

**Published:** 2024-03-13

**Authors:** Man Wang, Xiaofang Yan, Yanmei Li, Qian Li, Yingxia Xu, Jitian Huang, Juan Gan, Wenhan Yang

**Affiliations:** ^1^Department of Nutrition and Food Health, School of Public Health, Guangdong Pharmaceutical University, Guangzhou, Guangdong Province, China; ^2^Department of Child and Adolescent Health, School of Public Health, Guangdong Pharmaceutical University, Guangzhou, Guangdong Province, China; ^3^Guangzhou Baiyun District Maternal and Childcare Hospital, Guangzhou, Guangdong Province, China

**Keywords:** n-3, n-6, polyunsaturated fatty acids, depression, NHANES, PHQ-9, American, adult

## Abstract

**Background:**

Depression is associated with greater functional impairment and high societal costs than many other mental disorders. Research on the association between plasma polyunsaturated fatty acids (PUFAs) levels and depression have yielded inconsistent results.

**Objective:**

To evaluate whether plasma n-3 and n-6 PUFAs levels are associated with depression in American adults.

**Methods:**

A cross-sectional study included 2053 adults (aged ≥20 y) in the National Health and Nutrition Examination Survey (NHANES), 2011–2012. The level of plasma n-3 and n-6 PUFAs were obtained for analysis. Self-reported Patient Health Questionnaire-9 (PHQ-9) was used to identify the depression status. Binary logistic regression analysis was performed to evaluate the association between quartiles of plasma n-3 and n-6 PUFAs and depression after adjustments for confounders.

**Results:**

The study of 2053 respondents over 20 years of age with a weighted depression prevalence of 7.29% comprised 1,043 men (weighted proportion, 49.13%) and 1,010 women (weighted, 50.87%), with a weighted mean (SE) age of 47.58 (0.67) years. Significantly increased risks of depression over non-depression were observed in the third quartiles (OR = 1.65, 95% CI = 1.05–2.62) for arachidonic acid (AA; 20:4n-6); the third quartiles (OR = 2.20, 95% CI = 1.20–4.05) for docosatetraenoic acid (DTA; 22:4n-6); the third (OR = 2.33, 95% CI = 1.34–4.07), and highest quartiles (OR = 1.83, 95% CI = 1.03–3.26) for docosapentaenoic acid (DPAn-6; 22:5n-6); and the third (OR = 2.18, 95% CI = 1.18–4.03) and highest quartiles (OR = 2.47, 95% CI = 1.31–4.68) for docosapentaenoic acid (DPAn-3; 22:5n-3); the second (OR = 2.13, 95% CI = 1.24–3.66), third (OR = 2.40, 95% CI = 1.28–4.50), and highest quartiles (OR = 2.24, 95% CI = 1.08–4.69) for AA/docosahexaenoic acid (DHA; 22:6n-3) ratio compared with the lowest quartile after adjusting for confounding factors.

**Conclusion:**

Higher plasma levels of AA, DTA, DPAn-6, DPAn-3 PUFAs, and AA/DHA ratio may be potential risk factors for depression in US adults.

## 1 Introduction

Depression is a common and serious mental disorder and affects more than 280 million people globally (1). In fact, the World Health Organization ranked severe depression as the third cause of burden of disease worldwide as early as 2008 and projected that the disease will be the leading cause of disease burden worldwide by the year 2030 (2). In recent years, many studies have reported understanding the role of different influence factors, such as neurotransmitter, inflammatory markers and nutritional factors, to elucidate the underlying pathophysiology of depression in adults (3–5). Polyunsaturated fatty acids (PUFAs), as important nutrients, exhibit significant effects on the composition of the intestinal microflora as well as the function of the brain (6), and participate in numerous biological processes such as oxidation, neurotransmission, and inflammation (7, 8). Notably, PUFAs may play an important role in depression and its symptoms. Increasing evidence suggests that PUFAs could be associated with the pathophysiology of depression, as well as with the mechanisms underlying the therapeutic actions of antidepressants (9–11).

PUFAs are a class of fatty acids with two or more carbon–carbon double bonds (10). In human health, some PUFAs are considered essential nutrients, mainly including n-3 (primarily from fish, walnuts, wheat germ, and flaxseed) and n-6 PUFAs (primarily from refined vegetable oils such as corn, sunflower, and soybean), which cannot be synthesized in the body and must be obtained from dietary sources (8, 9). Various mental disorders such as Alzheimer disease (AD), dementia, attention-deficit/hyperactivity disorder (ADHD), autism spectrum disorder (ASD), schizophrenia, Bipolar disorders (BD) and depression have been suggested to be associated with altered levels and functions of PUFAs (6, 12–14). However, inconsistent conclusions remain, especially in the studies on depression.

The association between n-3 PUFAs and depression has been extensively investigated. Many studies showed that higher levels of n-3 PUFAs, mainly eicosapentaenoic acid (EPA; 20:5n-3) and DHA, were associated with a lower risk of depression (15–22). However, a few studies observed no apparent association with n-3 PUFAs (23–27), and, in particular, a recent longitudinal study did not support a protective effect of n-3 PUFAs on depression risk (28). In contrast to the cumulative evidence indicating the association of n-3 PUFAs with depression, the relationship between n-6 PUFAs and depression have received much less attention. Some studies found higher levels of n-6 PUFAs related to higher severity of depression, although the results of the relevant studies have been inconsistent (27, 29–32). Okubo’s study performed among Japanese breast cancer survivors indicated that a higher blood levels of n-6 PUFAs may increase the risk of depression, while the Avon Longitudinal Study (31) and Thesing’s study (33) reported no association in British and Dutch populations. Moreover, studies on the association of n-6/n-3 ratio and depression are also controversial. Some cross-sectional or longitudinal studies suggested a positive association (18, 34–36), whereas several other studies reported a negative association (37) or no association (25, 38).

Therefore, we comprehensively estimated the association of plasma n-3 and n-6 PUFAs and depression in a nationally representative sample of US adults aged 20 years and older. In order to provide a reference for elucidating the role of PUFAs on depression and a safer and more effective strategy to prevent or mitigate depressive symptoms in US adults.

## 2 Methods

### 2.1 Study design and sample

This is a cross-sectional study, done using the 2011–2012 cycle of The National Health and Nutrition Examination Survey (NHANES) data (39). NHANES is a stratified multistage probability sampling design to represent the noninstitutionalized civilian US population. Participants completed the survey through a computer-assisted personal interview and a medical examination at a mobile examination center (MEC). More detailed information regarding the survey design and data collection procedure are available elsewhere (40). The study protocol was approved by the National Center for Health Statistics research ethics review board. Written informed consent was obtained for all participants. The 2011–2012 cycle was utilized since all the main independent and dependent variables of interest were available only in this dataset (especially plasma PUFA). Since depression is more common in adult group (≥ 20 years), participants who were < 20 years were excluded for this study. Participants who did not fully respond to the depression screener questionnaire (PHQ-9) were excluded from the study (41). A total of 2053 adults from the United States were included in this study.

### 2.2 Determination and classification of depression status

Depression status were determined based on participant’s responses to the PHQ-9 questionnaire in the mental health-depression screener of questionnaire data of NHANES 2011–2012 cycle. PHQ-9 is the 9-item self-report depression scale that asks questions about the frequency of symptoms of depression over the past 2 weeks. Each item can be scored from 0 (not at all) to 3 (nearly every day) (42). The PHQ-9 score ranges from 0 to 27 and thus, classified in two categories. The individuals with PHQ-score < 9 were classified as “no or mild depression” and those with PHQ-score of 10 or more, were classified as “moderate to severe depression” (43, 44).

### 2.3 Assessment of plasma n-3 and n-6 PUFAs

Thirty fatty acids analyzed by means of gas chromatography–mass spectrometry and expressed in μmol/L were measured in serum with the goal of obtaining US reference ranges for most circulating fatty acids in a fasting subsample of participants. Briefly, total fatty acids were hexane-extracted from the matrix (100uL serum or plasma) along with an internal standard solution for fatty acid recovery. The extract was derivatized with pentafluorobenzyl bromide to form pentafluorobenzyl esters. The reaction mixture is injected onto a capillary gas chromatograph column (45). More details about plasma fatty acids profile analysis are available in the NHANES manual (40).

We chose all types of plasma n-3 and n-6 PUFAs tested in NHANES for our analysis. Moreover, we selected the most representative AA in n-6 PUFAs and the most representative DHA and EPA in n-3 PUFAs, and analyzed their ratios to reflect the different roles of n-3 and n-6 PUFAs in depression. Seventeen variables, total n-3 PUFAs, ALA, stearidonic acid (SDA; 18:4n-3), EPA, DHA, DPAn-3, total n-6 PUFAs, linoleic acid (LA; 18:2n-6), gamma-Linolenic acid (GLA; 18:3n-6), homo-gamma-Linolenic acid (DGLA; 20:3n-6), eicosadienoic acid (EDA; 20:2n-6), AA, DTA, DPAn-6, total n-6/n-3 ratio, AA/EPA ratio, and AA/DHA ratio, were evaluated in the present study.

### 2.4 Assessment of life’s essential 8

LE8 scoring algorithm consists of 4 health behaviors (diet, physical activity, nicotine exposure, and sleep health) and 4 health factors (body mass index [BMI], blood pressure, glucose, lipids) (46). This study selected 5 individual LE8 metrics (i.e., blood pressure, lipids, glucose, BMI and nicotine exposure) that were more relevant to the study variables (Table 1). LE8 has great potential to assess and promote cardiovascular health (CVH) across life course. CVH is associated with cardiovascular disease (CVD), as well as non-CVD outcomes such as cognitive impairment and depression (47). CVH-related factors routinely collected (i.e., BMI, smoking, hypertension, hypercholesterolemia, and diabetes) were more relevant to our study variables and could be used to accurately estimate individuals’ overall CVH across time even when LE8 metrics are incomplete (48).

**Table 1:** Scoring criteria of CVH-related factors metrics based on life’s essential 8.

Metric	Points and criteria
Blood pressure	100: SBP < 115 mmHg and DBP < 75 mmHg 75: SBP 115-124 mmHg and DBP < 75 mmHg 50: SBP 125-134 mmHg or DBP: 75-84 mmHg 25: SBP 135-154 mmHg or DBP 85-94 mmHg 0: SBP ≥ 155 mmHg or DBP ≥ 95 mmHg (Subtract 20 points if treated level)
HbA1c	100: No history of diabetes and HbA1c < 5.7% 60: No diabetes and HbA1c 5.7–6.4% 40: Diabetes with HbA1c < 7.0% 30: Diabetes with HbA1c 7.0–7.9% 20: Diabetes with HbA1c 8.0–8.9% 10: Diabetes with HbA1c 9.0–9.9% 0: Diabetes with HbA1c ≥ 10.0%
Blood lipids	100: Non-HDL cholesterol <130 mg/dL 60: Non-HDL cholesterol 130–159 mg/dL 40: Non-HDL cholesterol 160–189 mg/dL 20: Non-HDL cholesterol 190–219 mg/dL 0: Non-HDL cholesterol ≥220 mg/dL (Subtract 20 points if treated level)
Nicotine exposure	100: Never smoker 75: Former smoker, quit ≥5 year 50: Former smoker, quit 1–5 year 25: Former smoker, quit <1 year 0: Current smoker (Subtract 20 points if living with active indoor smoker in home)
BMI	100: < 25 kg/m2 70: 25.0–29.9 kg/m2 30: 30.0–34.9 kg/m2 15: 35.0–39.9 kg/m2 0: ≥ 40.0 kg/m2

BMI, body mass index; CVH, cardiovascular health; DBP, diastolic blood pressure; HbA1c, glycohemoglobin; HDL, high-density lipoprotein; SBP, systolic blood pressure.

### 2.5 Other covariates

Based on existing literatures on PUFAs and depression, age, sex, race/ethnicity (Non-Hispanic White, Non-Hispanic Black, Mexican American, Other Hispanic and Other race), educational level and poverty income ratio (PIR) were included in this study as other potential confounders (28, 31, 49). Highest educational level was categorized into 3 levels: (1) Less than high school/general education development (GED), (2) High school graduate/GED or equivalent and (3) higher than high school graduate/GED. PIR represent the ratio of family or unrelated individual income to their appropriate poverty threshold (50). It was categorized into three groups: (1) low: ≤ 1.3, (2) medium: 1.3–3.5, (3) high: > 3.5 (51).

### 2.6 Statistical analysis

Following the NHANES analytic guidelines, all analyses in this study accounted for sample weights, clustering, and stratification to generate nationally representative estimates (39).

Means and percentages of baseline characteristics were compared using t-tests for continuous variables and χ^2^ test for categorical variables. PUFAs were grouped into quartiles and analyzed for the inter-quartile group trend. The lowest quartile (first quartile) was defined as the reference group in each model. Binary logistic regression analysis was used to assess the relation between quartiles of plasma PUFAs level and depression. These regression analyses employed 4 sets of covariates: model 1 was crude model; model 2 adjusted for age, sex and race/ethnicity; Model 3 adjusted the ratio of family income to poverty and personal highest education level on the basis of Model 2; and Model 4 additionally adjusted cardiovascular health on the basis of Model 3.

All statistical analyses were performed using SAS software, version 9.4 (SAS Institute, Cary, NC, United States). Statistical significance was set at 2-sided *p* < 0.05.

This analysis was conducted using publicly available, deidentified data and was not subject to review by the Guangdong Pharmaceutical University’s institutional review board.

## 3 Results

### 3.1 Demographic characteristics of study population

A total of 2053 US adults aged 20–80 who participated in NHANES 2011–2012 enrolled into the study (Table 2). 7.29% (weighted) subjects had a PHQ-9 score ≥ 10 and were categorized as depressed (moderate to severe depression), and 92.71% (weighted) subjects with PHQ-9 score < 10 were categorized as not depressed. The weighted mean (standard error, SE) age of the study participants was 47.58 (0.67) years, and male and female participants were almost equally distributed gender wise. Among the participants, 821 (68.10%) were non-Hispanic white, 477 (11.21%) were non-Hispanic black, 213 (7.42%) were Mexican-American, 209 (5.89%) were Other Hispanic, and 333 (7.37%) were other race/ethnicity. About 83.59% of respondent had completed at least high school/GED. Among adults, 22.40% had low income (PIR ≤ 1.3), 33.66% had medium income (1.3 < PIR ≤ 3.5) and 37.89% high income (3.5 < PIR). Average CVH score was 70.54 (0.82) and the percentages of low, moderate, and high CVH were17.62, 52.20, and 30.18%, respectively. Significantly participants with lower PIR and CVH level were more likely to have depression.

**Table 2:** Descriptive characteristics of all Participants for total sample and by depression level groups among US adults in NANES, 2011–2012.

Characteristics	Total	Without depression	Depression	χ^2^/ t	*p* value
No. of participants (%)	2053(100)	1890(92.71)	163(7.29)	–	–
Age, y [mean(SE)]	47.58(0.67)	47.70(0.72)	46.03(0.93)	−1.41	0.18
Sex, N (%)					
Male	1,043(49.13)	983(49.99)	60(38.24)	3.59	0.06
Female	1,010(50.87)	907(50.01)	103(61.76)		
Race/ethnicity, N (%)					
Mexican American	213(7.42)	198(7.55)	15(5.86)	0.73	0.57
Other Hispanic	209(5.89)	187(5.66)	22(8.78)		
Non-Hispanic White	821(68.10)	746(68.08)	75(68.34)		
Non-Hispanic Black	477(11.21)	443(11.36)	34(9.33)		
Others	333(7.37)	316(7.35)	17(7.70)		
Education level, N (%)					
< High school	457(16.39)	402(15.82)	55(23.68)	2.51	0.08
High school	433(20.04)	389(19.48)	44(27.24)		
> High school	1,162(63.55)	1,098(64.69)	64(49.08)		
Missing	1(0.01)	1(0.01)	0(0)		
Ratio of family income to poverty, N (%)					
≤ 1.3	655(22.40)	557(20.60)	98(45.36)	13.78	< 0.001*
1.3–3.5	659(33.66)	617(33.54)	42(35.29)		
> 3.5	573(37.89)	560(39.79)	13(13.62)		
Missing	166(6.05)	156(6.07)	10(5.73)		
Total CVH-related factors score, [mean(SE)]					
Body mass index score	61.16(1.56)	61.48(1.63)	57.03(3.34)	−1.15	0.26
Nicotine exposure score	73.15(1.40)	74.85(1.34)	51.54(4.56)	−5.19	< 0.001*
Blood lipids score	64.28(1.14)	64.90(1.21)	56.48(2.70)	−2.62	0.02*
HbA1c score	84.77(0.97)	84.99(1.01)	81.98(2.39)	−1.23	0.23
Blood pressure score	66.71(1.20)	66.84(1.21)	65.10(2.49)	−0.73	0.48
Total score	70.54(0.82)	71.17(0.83)	62.60(1.38)	−4.47	0.003*
Cardiovascular health^a^, N (%)					
Low	450(17.62)	390(16.98)	60(25.75)	4.44	0.01*
Moderate	1,041(52.20)	967(51.94)	74(55.62)		
High	562(30.18)	533(31.08)	29(18.64)		

*p < 0.05.

a Low CVH was defined as a CVH-related factors score of 0 to 49, moderate CVH of 50–79, and high CVH of 80–100.

### 3.2 The association between PUFAs and depression: univariate analyses

The univariate analyses of PUFAs as continuous variables indicated that there was an association between part of plasma PUFAs levels and depression among US adults (*p* < 0.05). The weighted mean (SE) of those plasma PUFAs for GLA, DGLA, EDA, EPA, DTA, DHA, DPAn-6, DPAn-3 and AA/DHA ratio were 64.67 (1.46), 167.35 (2.85), 23.92 (0.47), 70.96 (1.99), 27.36 (0.58), 162.61 (4.74), 20.78 (0.43), 53.69 (0.78), and 6.33 (0.16) respectively (Table 3); PUFAs as categorical variables indicated that there were a difference in quartiles of plasma PUFAs levels of DTA, DHA, DPAn-6, total n-6/n-3 ratio and AA/DHA ratio among US adults with depression and non-depression (*p* < 0.05; Table 4).

**Table 3:** Univariate analyses results of the relationship between continuous plasma n-3 and n-6 polyunsaturated fatty acids and depression in American adults: NHANES, 2011–2012.

PUFA, μmol/L, [mean(SE)]	Total	Without depression	Depression	t	*p* value
LA(18:2n-6)	3848.96(48.05)	3837.00(48.93)	3999.65(112.63)	1.42	0.17
GLA(18:3n-6)	64.67(1.46)	64.09(1.38)	72.00(3.31)	3.28	0.004*
ALA(18:3n-3)	93.42(2.02)	93.09(1.98)	97.64(4.25)	1.41	0.18
SDA(C18:4n-3)	4.26(0.16)	4.19(0.16)	5.11(0.56)	1.99	0.06
DGLA(20:3n-6)	167.35(2.85)	166.10(2.95)	184.48(7.61)	2.30	0.03*
EDA(20:2n-6)	23.92(0.47)	23.77(0.44)	25.91(1.34)	2.46	0.03*
AA(20:4n-6)	891.57(10.22)	888.89(9.83)	925.38(22.32)	1.96	0.07
EPA(20:5n-3)	70.96(1.99)	71.83(2.03)	59.95(4.01)	−2.21	0.04*
DTA(22:4n-6)	27.36(0.58)	27.08(0.54)	31.05(1.35)	4.90	< 0.001*
DHA(22:6n-3)	162.61(4.74)	162.61(4.74)	146.53(5.40)	−3.07	0.007*
DPA6(22:5n-6)	20.78(0.43)	20.56(0.41)	23.57(0.95)	4.95	< 0.001*
DPA3 (22:5n-3)	53.69(0.78)	53.44(0.76)	56.92(1.82)	2.33	0.03*
Total n-3	386.21(6.28)	387.54(6.60)	369.43(11.55)	−1.21	0.24
Total n-6	5049.47(64.66)	5033.69(66.24)	5268.74(142.14)	1.60	0.13
n-6/n-3 ratio	14.49(0.31)	14.46(0.32)	14.95(0.32)	1.49	0.15
AA/EPA	18.05(0.50)	17.90(0.46)	19.92(1.41)	1.87	0.08
AA/DHA	6.33(0.16)	6.29(0.16)	6.86(0.20)	3.33	0.004*

*p < 0.05.

**Table 4:** Univariate analyses results of the relationship between quartiles of plasma n-3 and n-6 polyunsaturated fatty acids and depression in American adults: NHANES, 2011–2012.

PUFA, μmol/L, N (%)	Total	Without depression	Depression	χ^2^	*p* value
LA(18:2n-6)	1,593(100)	1,467(92.52)	126(7.48)		
[1430.00, 2989.84]	373(24.61)	341(24.57)	32(25.06)	0.23	0.88
[2989.84, 3451.86]	391(25.28)	365(25.51)	26(22.52)		
[3451.86, 4026.28]	413(25.00)	383(25.10)	30(23.72)		
[4026.28, 15900.00]	416(25.11)	378(24.82)	38(28.70)		
GLA(18:3n-6)	1,549(100)	1,428(92.80)	121(7.20)		
[6.61, 30.22]	371(24.99)	341(25.22)	30(21.93)	0.37	0.77
[30.22, 42.89]	368(24.72)	343(24.73)	25(24.55)		
[42.89, 64.91]	411(25.29)	381(25.41)	30(23.70)		
[64.91, 391.00]	399(25.01)	363(24.63)	36(29.82)		
ALA(18:3n-3)	1,592(100)	1,466(92.52)	126(7.48)		
[16.80, 50.91]	379(24.98)	346(24.84)	33(26.69)	0.36	079
[50.91, 68.27]	384(24.89)	359(25.22)	25(20.89)		
[68.27, 96.45]	414(25.08)	384(25.19)	30(23.72)		
[96.45, 803.00]	415(25.05)	377(24.75)	38(28.70)		
SDA(C18:4n-3)	1798(100)	1,648(92.48)	150(7.52)		
[0.17, 2.05]	500(24.74)	463(25.11)	37(20.28)	1.98	0.11
[2.05, 3.18]	475(25.22)	438(25.53)	37(21.48)		
[3.18, 5.13]	408(24.89)	376(25.06)	32(22.89)		
[5.13, 104.00]	415(25.13)	371(24.30)	44(35.35)		
DGLA(20:3n-6)	1934(100)	1787(93.16)	147(6.84)		
[29.00, 122.75]	560(24.02)	529(24.33)	31(19.83)	2.20	0.09
[122.75, 161.13]	527(25.91)	492(26.49)	35(18.02)		
[161.13, 197.92]	393(24.29)	358(24.08)	35(27.22)		
[197.92, 465.00]	454(25.77)	408(25.10)	46(34.92)		
EDA(20:2n-6)	1826(100)	1,679(92.70)	147(7.30)		
[8.75, 18.09]	439(24.32)	410(22.58)	29(23.86)	0.76	0.52
[18.09, 22.18]	444(24.74)	411(23.35)	33(19.05)		
[22.18, 27.71]	468(25.86)	432(24.11)	36(23.99)		
[27.71, 134.00]	475(25.07)	426(22.66)	49(33.09)		
AA(20:4n-6)	2041(100)	1878(92.66)	163(7.34)		
[187.00, 713.84]	533(24.96)	500(25.51)	33(18.09)	1.81	0.14
[713.84, 868.54]	515(24.97)	476(24.83)	39(26.75)		
[868.54, 1040.25]	492(25.05)	450(24.60)	42(30.71)		
[1040.25, 2570.00]	501(25.02)	452(25.07)	49(24.44)		
EPA(20:5n-3)	2041(100)	1878(92.66)	163(7.34)		
[8.76, 35.96]	551(24.89)	508(24.84)	43(25.55)	1.24	0.29
[35.96, 53.19]	492(24.87)	450(24.72)	42(26.76)		
[53.19, 82.38]	512(25.14)	465(24.64)	47(31.44)		
[82.38, 1090.00]	486(25.10)	455(25.80)	31(16.25)		
DTA(22:4n-6)	1975(100)	1822(92.96)	153(7.26)		
[1.71, 20.23]	543(24.87)	522(25.82)	21(12.76)	4.32	0.005*
[20.23, 25.63]	489(24.97)	485(25.49)	31(18.42)		
[25.63, 32.48]	461(24.71)	412(23.94)	49(34.66)		
[32.48, 204.00]	482(25.44)	430(24.76)	52(34.16)		
DHA(22:6n-3)	2032(100)	1870(92.68)	162(7.32)		
[25.70, 107.61]	449(24.80)	245(25.10)	32(23.58)	3.76	0.01*
[107.61, 144.05]	480(25.17)	247(23.14)	55(44.07)		
[144.05, 195.42]	519(24.82)	289(25.38)	32(14.75)		
[195.42, 918.00]	584(25.22)	326(26.38)	43(17.61)		
DPA6(22:5n-6)	1982(100)	1828(92.72)	154(7.28)		
[2.18, 14.56]	485(24.89)	466(25.78)	19(13.51)	9.34	< 0.001*
[14.56, 19.22]	489(24.94)	461(25.85)	28(13.33)		
[19.22, 25.15]	492(24.91)	444(23.87)	48(38.03)		
[25.15, 126.00]	516(25.27)	457(24.49)	59(35.12)		
DPA3 (22:5n-3)	1998(100)	1844(92.82)	154(7.18)		
[12.60, 40.17]	562(24.77)	534(25.55)	28(14.67)	2.11	0.10
[40.17, 49.70]	497(25.21)	460(25.22)	37(25.10)		
[49.70, 62.06]	451(24.85)	412(24.63)	39(27.80)		
[62.06, 229.00]	488(25.16)	438(24.60)	50(32.43)		
Total n-3	1,369(100)	1,260(92.56)	368(25.03)		
[91.58, 268.26]	310(24.82)	284(25.26)	26(19.34)	1.35	0.26
[268.26, 344.47]	355(25.08)	330(24.49)	25(32.39)		
[344.47, 441.24]	336(25.07)	303(24.49)	33(32.35)		
[441.24, 1917.50]	368(25.03)	343(25.76)	25(15.93)		
Total n-6	1,323(100)	1,226(93.55)	97(6.45)		
[2913.92, 4193.90]	321(24.89)	300(25.47)	21(16,54)	0.84	0.47
[4193.90, 4662.68]	328(25.09)	307(25.22)	21(23.24)		
[4662.68, 5296.08]	332(25.00)	308(24.59)	24(30.94)		
[5296.08, 17189.00]	342(25.01)	311(24.72)	31(29.28)		
n-6/n-3 ratio	1,198(100)	1,107(93.52)	91(6.48)		
[2.25, 11.38]	311(24.88)	290(25.05)	21(22.30)	2.85	0.04*
[11.38, 14.17]	312(25.10)	287(25.12)	25(24.84)		
[14.17, 16.75]	296(25.02)	269(24.12)	27(37.95)		
[16.75, 28.60]	279(25.01)	261(25.71)	18(14.91)		
AA/EPA	2040(100)	1877(92.65)	163(7.35)		
[0.36, 11.19]	494(24.79)	468(25.62)	26(14.30)	2.35	0.07
[11.19, 16.85]	500(25.14)	450(24.45)	50(33.87)		
[16.85, 23.24]	468(25.04)	435(25.22)	33(22.71)		
[23.24, 73.13]	578(25.03)	524(24.71)	54(29.12)		
AA/DHA	2032(100)	1870(92.68)	162(7.32)		
[0.77, 4.47]	575(25.00)	545(26.11)	30(10.88)	7.44	< 0.001*
[4.47, 6.15]	532(24.96)	489(24.88)	43(26.08)		
[6.15, 7.73]	487(24.88)	437(24.31)	50(32.16)		
[7.73, 18.91]	438(25.16)	399(24.71)	39(30.88)		

*p < 0.05.

### 3.3 The association between PUFAs and depression: multiple regression analysis

Table 5 present the OR and 95% CI of depression for quartiles of PUFAs concentrations, using the lowest quartile category as the reference. After controlling for age, sex, race/ethnicity, ratio of family income to poverty, personal highest education level and CVH in the fully adjusted model (model 4), there was a positive relationship between depression and PUFAs in the third quartiles (OR = 1.65, 95% CI = 1.05–2.62) for AA; the third quartiles (OR = 2.20, 95% CI = 1.20–4.05) for DTA; the third (OR = 2.33, 95% CI = 1.34–4.07), and highest quartiles (OR = 1.83, 95% CI = 1.03–3.26) for DPAn-6; and the third (OR = 2.18, 95% CI = 1.18–4.03) and highest quartiles (OR = 2.47, 95% CI = 1.31–4.68) for DPAn-3; 22:5n-3; the second (OR = 2.13, 95% CI = 1.24–3.66), third (OR = 2.40, 95% CI = 1.28–4.50), and highest quartiles (OR = 2.24, 95% CI = 1.08–4.69) for AA/DHA ratio (Table 5).

**Table 5:** Association of plasma n-3 and n-6 polyunsaturated fatty acids levels with depression in American adults: NHANES, 2011–2012^a^.

PUFA, μmol/L, N	Model 1	Model 2	Model 3	Model 4	*p* trend^b^
	OR (95% CI)	OR (95% CI)	OR (95% CI)	OR (95% CI)	
AA(20:4n-6)					
[187.00, 713.84](*n* = 533)	Ref	Ref	Ref	Ref	0.86
[713.84, 868.54](*n* = 515)	1.52(0.79, 2.94)	1.60(0.85, 3.02)	1.58(0.82, 3.03)	1.46(0.76, 2.80)	
[868.54, 1040.25](*n* = 492)	1.76(1.09, 2.86)	1.88(1.16, 3.05)	1.93(1.25, 3.00)	1.65(1.05, 2.62)	
[1040.25, 2570.00](*n* = 501)	1.38(0.88, 2.15)	1.51(0.91, 2.50)	1.41(0.77, 2.58)	1.15(0.66, 2.02)	
DTA(22:4n-6)					
[1.71, 20.23](*n* = 543)	Ref	Ref	Ref	Ref	0.15
[20.23, 25.63](*n* = 489)	1.46(0.55, 3.91)	1.54(0.59, 4.01)	1.40(0.50, 3.95)	1.25(0.44, 3.57)	
[25.63, 32.48](*n* = 461)	2.93(1.65, 5.21)	3.19(1.84, 5.55)	2.63(1.50, 4.61)	2.20(1.20, 4.05)	
[32.48, 204.00](*n* = 482)	2.79(1.44, 5.42)	3.18(1.72, 5.87)	2.63(1.22, 5.70)	2.13(0.96, 4.70)	
DPA6(22:5n-6)					
[2.18, 14.56](*n* = 485)	Ref	Ref	Ref	Ref	0.06
[14.56, 19.22](*n* = 489)	0.98(0.62, 1.57)	0.95(0.59, 1.53)	0.85(0.48, 1.52)	0.75(0.40, 1.41)	
[19.22, 25.15](*n* = 492)	3.04(1.73, 5.35)	3.02(1.71, 5.33)	2.68(1.57, 4.57)	2.33(1.34, 4.07)	
[25.15, 126.00](*n* = 516)	2.74(1.82, 4.12)	2.71(1.79, 4.11)	2.26(1.30, 3.91)	1.83(1.03, 3.26)	
DPA3 (22:5n-3)					
[12.60, 40.17](*n* = 562)	Ref	Ref	Ref	Ref	0.08
[40.17, 49.70](*n* = 497)	1.73(0.85, 3.55)	1.99(0.97, 4.11)	2.18(1.09, 4.36)	1.95(0.96, 3.95)	
[49.70, 62.06](*n*= 451)	1.97(1.02, 3.81)	2.39(1.19, 4.79)	2.46(1.37, 4.42)	2.18(1.18, 4.03)	
[62.06, 229.00](*n* = 488)	2.30(1.35, 3.92)	2.84(1.71, 4.73)	2.95(1.62, 5.39)	2.47(1.31, 4.68)	
AA/DHA ratio					
[0.77, 4.47](*n* = 575)	Ref	Ref	Ref	Ref	0.12
[4.47, 6.15](*n* = 532)	2.52(1.52, 4.17)	2.58(1.55, 4.29)	2.24(1.31, 3.85)	2.13(1.24, 3.66)	
[6.15, 7.73](*n* = 487)	3.18(1.89, 5.35)	3.42(1.97, 5.93)	2.54(1.35, 4.80)	2.40(1.28, 4.50)	
[7.73, 18.91](*n* = 438)	3.00(1.68, 5.36)	3.42(1.74, 6.74)	2.40(1.13, 5.10)	2.24(1.08, 4.69)	

Model 1: crude model. Model 2: adjusted for age, sex and race/ethnicity. Model 3: Model 2 plus ratio of family income to poverty, personal highest education level. Model 4: Model 3 plus CVH.

a Only the statistically significant results were shown after controlling all potential confounders.

b Test for trend based on the variable containing a median value for each quartile.

### 3.4 The association between PUFAs and depression: stratified analyses

Adjusted model was performed to observe the association between the PUFAs and depression by keeping all other study variables constant (Table 6). With increased plasma levels of AA, DPAn-3 and AA/DHA ratio as well as decreased plasma levels of DHA, women have an increased risk of depression. The risk of depression increases with increased plasma levels of AA and DPAn-6 among ≥60 years age group. Participants aged between 40 and 59 years old were more likely to develop depression due to elevated plasma DPAn-3 levels. Furthermore, elevated plasma AA/DHA levels in the 20–39 years age group may be a risk factor for depression.

**Table 6:** Stratified analyses of the association of plasma n-3 and n-6 polyunsaturated fatty acids levels with depression in American adults: NHANES, 2011–2012.^a^

PUFA, μmol/L, N	Male	OR (95% CI)	*p* trend^b^	Female	OR (95% CI)	*p* trend^b^
AA(20:4n-6)	*n* = 1,039			*n* = 1,002		
	[187.00, 711.80](*n* = 290)	Ref	0.68	[295.00, 718.63](*n* = 243)	Ref	0.99
	[711.80, 856.29](*n* = 273)	0.92(0.37, 2.29)		[718.63, 878.13](*n* = 242)	1.99(1.00, 3.93)	
	[856.29, 1033.88](*n* = 242)	1.26(0.48, 3.26)		[878.13, 1053.00](*n* = 250)	2.44(1.36, 4.39)	
	[1033.88, 2570.00](*n* = 234)	0.84(0.34, 2.09)		[1053.00, 2560.00](*n* = 267)	1.41(0.65, 3.05)	
DHA(22:6n-3)	*n* = 1,033			*n* = 999		
	[34.30, 99.89](*n* = 273)	Ref	0.30	[25.70, 116.76](*n* = 176)	Ref	0.005*
	[99.89, 136.00](*n* = 253)	2.06(0.53, 8.08)		[116.76, 152.40](*n* = 227)	2.24(0.86, 5.88)	
	[136.00, 187.07](*n* = 258)	1.11(0.25, 4.90)		[152.40, 205.87](*n* = 261)	0.50(0.28, 0.87)	
	[187.07, 918.00](*n* = 249)	1.96(0.91, 4.22)		[205.87, 799.00](*n* = 335)	0.49(0.25, 0.96)	
DPA3 (22:5n-3)	*n* = 1,014			*n* = 984		
	[12.6 40.79](*n* = 271)	Ref	0.15	[15.10, 39.97](*n* = 291)	Ref	0.13
	[40.79, 49.90](*n* = 259)	1.66(0.49, 5.62)		[39.97, 49.39](*n* = 238)	2.06(1.00, 4.25)	
	[49.90, 61.18](*n* = 240)	2.32(0.78, 6.86)		[49.39, 62.65](*n* = 211)	2.08(0.96, 4.51)	
	[61.18, 229.00](*n* = 244)	2.40(0.78, 7.42)		[62.65, 198.00](*n* = 244)	2.39(1.28, 4.47)	
AA/DHA	*n* = 1,033			*n* = 999		
	[0.92, 4.82](*n* = 266)	Ref	0.17	[0.77, 4.36](*n* = 309)	Ref	0.004*
	[4.82, 6.52](*n* = 254)	2.38(0.81, 7.00)		[4.36, 5.98](*n* = 278)	1.94(0.77, 4.88)	
	[6.52, 8.12](*n* = 244)	2.07(0.73, 5.84)		[5.98, 7.28](*n* = 243)	2.32(1.12, 4.81)	
	[8.12, 18.91](*n* = 269)	0.89(0.19, 4.19)		[7.28, 17.40](*n* = 169)	4.09(2.14, 7.84)	

*p < 0.05.

a Analyses were adjusted for age, sex, education, race/ethnicity, ratio of family income to poverty and CVH except the stratification variable, and only the statistically significant results were shown after controlling all potential confounders.

b Test for trend based on the variable containing a median value for each quartile.

## 4 Discussion

Our findings suggest that there was a significant association between plasma PUFAs with moderate or severe level of depression in American adults after controlling all potential confounders. There were higher odds of developing depression among people who have higher plasma levels of n-6 PUFAs (AA, DTA, and DPAn-6), n-3 PUFAs (DPAn-3) and AA/DHA ratio. Our analysis consolidated the risk role of higher plasma levels of AA and AA/DHA ratio in depression, and was the first to report a positive association between higher plasma DTA, DPAn-6, and DPAn-3 levels and depression in America adult.

The weighted prevalence of depression among subjects was 7.29% in our study. A report from NHANES, 2009–2012 data set has indicated that 7.6% of Americans aged 12 and over had depression (52). The incidence rate is approximately the same as our study. We assessed the participants’ blood lipid levels by LE8, and the blood lipids score was 64.28 (1.14). In Lili Wang’s research on the associations between LE8 and non-alcoholic fatty liver disease among US adults, the blood lipids score was 67.0 (2.7) which is also roughly in the same way as in our study (53). Overall, our findings suggest that the prevalence of depression and blood lipid levels in our study are roughly in line with the other relevant studies.

Our data provided evidence for existence of associations of depression with higher plasma levels of AA and AA/DHA ratio in American adult sample, which is consistent with some earlier research (54–56). This may reflect the opposing effects of AA and DHA on depression, although the association between plasma DHA and depression did not persist after controlling for all the potential confounding factors. AA, is an abundant n-6 PUFA in the membrane phospholipids, where it is stored in the sn-2 position of phospholipids (57). AA-derived eicosanoids from cyclooxygenase (COX) pathways or lipoxygenase (LOX) pathways are important lipid mediators involved in a number of physiological and pathophysiological processes ranging from inflammation, allergic responses, blood lipid regulation, to cell metabolism (58). Cyclooxygenases participate in the production of pro-inflammatory eicosanoids, such as prostaglandins and thromboxanes. On the other hand, lipoxygenases generate both leukotrienes and anti-inflammatory eicosanoids like lipoxins. Consequently, an imbalance between these AA-derived eicosanoids has been suggested as a contributing factor to inflammatory effects. Inflammatory processes within the central nervous system (CNS) are essential for the development of brain pathologies, including depression (59). Increased levels of inflammatory cytokines have been reported in depressed patients (60), and found to promote abnormalities in neurotransmitter metabolism and neuroendocrine function, which are related to the pathophysiology of depression (61). Whereas DHA, an n-3 PUFA, has a certain anti-inflammatory effect through competition with AA. Furthermore, DHA also has a neuroprotective effect against decreased neurogenesis and increased neuronal apoptosis (22). Increased plasma levels of AA and/or decreased DHA may cause a disproportionate ratio of n-6 to n-3 and lead to depression through increased inflammatory processes.

This study also found elevated plasma level of other n-6 PUFAs such as DTA and DPAn-6 in patients with moderate to severe depression. DTA, a 2-carbon elongation product of AA, can be desaturated to generate DPAn-6 (62). Previous studies have shown that DTA can impair neurobehavioral development by increasing reactive oxidative species production in Caenorhabditis elegans (63), and higher DPAn-6 status also showed an association with worse mental health in older adults with mild cognitive impairment (64) and children and adolescents with bipolar disorder (65). The Western diet, which is high in processed foods with fat and sugar, is increasing the risk of depression (66). In addition, reactive oxygen species and inflammatory cytokines that could be induced by unreasonable intake of PUFAs have the importance in the progression of depression (67). Previous studies have shown that some n-6 PUFAs are able to activate inflammatory responses and lead to the accumulation of reactive oxygen species and pro-inflammatory factors (68, 69). These alterations might affect the integrity of the cell membrane, leading to alteration of intestinal flora, systemic inflammation and abnormal neurotransmitter transport in the brain (6, 70). In summary, abnormalities in plasma n-6 PUFAs metabolism may be involved in the progress of depression.

The DPAn-3 is less studied as a new player in the n-3 PUFAs family, compared to its counterparts EPA and DHA. The literature on DPAn-3 is limited, however most of the available data suggests it has beneficial health effects which is contrary to our found that higher DPAn-3 was associated with depression. Our result agrees with a minority of reports concerning DPAn-3 (71). More research remains to be done to further investigate the biological effects of this DPAn-3.

There were also some studies that did not observe an association between plasma PUFAs levels and depression (31, 33, 49, 72, 73). But most of these studies have focused only on the total n-3 PUFAs and/or the total n-6 PUFAs, or the canonical fatty acids among them. A longitudinal study in middle-aged Finnish men did not find evidence that serum polyunsaturated fatty acids would be associated with risk of depression (38). The composition ratio of sex may partially influence results. According to previous studies of dietary intervention in Western countries, the association appears to be observed more in women than in men (21, 74). We also observed a significant association for AA, DHA, DPAn-3, and AA/DHA ratio in women through a sub-analysis stratified by sex. However, this finding should be interpreted with caution in light of the source of PUFAs.

One of the strengths of this study is the use of data from a nationally representative survey with a large sample size from a multiracial/multiethnic population. Even a large number of analyses have been conducted to assess the role of PUFAs in depression from multiple perspectives, especially at the supplement and diet levels, but only a few studies with small sample sizes have examined the association between depression and plasma PUFAs. To our knowledge, our study is the first observational study to examine the association between plasma PUFAs and depression among American adult using part of the LE8 indicators as the primary covariate.

Another strength of this study is that we used plasma PUFAs, which better reflects tissue levels of PUFAs than the more subjective measures of the Food Frequency Questionnaires (FFQ) and food records. Several limitations also need to be acknowledged. A major limitation is that this study is only a cross-sectional exploratory investigation, so a causal relationship cannot be concluded. Additionally, the PHQ-9 items measure only a subset of the symptoms of depression. Apart from the nine symptoms of the PHQ, other depressive symptoms were not included in this study which may be counted as a limitation.

## 5 Conclusion

In conclusion, this population-based cross-sectional study examining the association between plasma PUFAs and depression risk in American adults revealed an association between higher plasma levels of AA, DTA, DPAn-6, DPAn-3 and AA/DHA ratio and increased risk of depression. The complexity of PUFAs metabolism, interactions and competition between n-3 PUFAs and n-6 PUFAs and the mechanism by which these may influence depression requires continued study.

## Data availability statement

Publicly available datasets were analyzed in this study. This data can be found here: https://www.cdc.gov/.

## Ethics statement

The studies involving humans were approved by National Center for Health Statistics research ethics review board. The studies were conducted in accordance with the local legislation and institutional requirements. The participants provided their written informed consent to participate in this study.

## Author contributions

MW: Conceptualization, Data curation, Formal analysis, Investigation, Methodology, Software, Writing – original draft, Writing – review & editing. XY: Data curation, Formal analysis, Software, Writing – review & editing. YL: Data curation, Methodology, Writing – review & editing. QL: Software, Supervision, Writing – review & editing. YX: Data curation, Project administration, Writing – review & editing. JH: Data curation, Methodology, Writing – review & editing. JG: Funding acquisition, Project administration, Resources, Validation, Visualization, Writing – review & editing. WY: Conceptualization, Formal analysis, Funding acquisition, Project administration, Resources, Validation, Visualization, Writing – review & editing.
